# Enhancing dosimetric precision in the treatment of cancerous tumors: Gamma Index validation and Monte Carlo simulations of 6 and 12 megavoltage photon beams from Varian Medical linear accelerators

**DOI:** 10.3389/fonc.2025.1573166

**Published:** 2025-08-20

**Authors:** Ali H. D. Alshehri

**Affiliations:** Department of Radiological Sciences, College of Applied Medical Sciences, Najran University, Najran, Saudi Arabia

**Keywords:** percentage of depth dose (PDD), GAMOS/GATE codes (Monte Carlo), LINAC, Varian, radiotherapy

## Abstract

**Introduction:**

The accuracy of dose delivery in radiotherapy is paramount to maximize tumor control while minimizing damage to surrounding healthy tissues. This study presents a comprehensive analysis of gamma index validation in the treatment of cancerous tumors using Monte Carlo simulations with GAMOS and GATE codes on a Varian medical linear accelerator. By leveraging the MC method’s robust statistical capabilities, the precision of dose distributions in external radiotherapy is aimed to be enhanced. The study specifically evaluates the effects of different field sizes and percentage depth dose (PDD) to provide a thorough validation framework.

**Methods:**

The GAMOS and GATE codes were implemented to simulate dose distributions within various phantom models, including water and anthropomorphic phantoms. These simulations were conducted using a Varian linear accelerator with a 6 and 12 megavoltage photon beams. The dose distributions obtained from the simulations were then compared against those calculated by the treatment planning system (TPS) using the gamma index method with 3%/3mm criteria.

**Results and discussion:**

The results demonstrated a high degree of accuracy in the simulated dose distributions, with gamma index pass rates exceeding 94% for most configurations. The comparative analysis between GAMOS and GATE showed consistent performance, with minor deviations attributable to differences in the underlying simulation algorithms. Furthermore, the study revealed significant insights into the impact of varying field sizes on dose distribution accuracy. The PDD analysis indicated that both GAMOS and GATE could reliably reproduce the TPS-calculated dose profiles, with deviations within clinically acceptable limits. These findings underscore the potential of MC simulations to improve the accuracy and reliability of radiotherapy treatment plans. By validating the gamma index for different field sizes and PDD, this study provides a robust framework for enhancing treatment efficacy and patient safety in clinical practice. The integration of GAMOS and GATE in routine clinical workflows could lead to more precise and individualized radiotherapy treatments, ultimately improving patient outcomes.

## 1 Introduction

Radiotherapy is a pivotal modality in cancer treatment, with the primary goal of delivering a precise dose of radiation to malignant tissues while minimizing exposure to surrounding healthy structures (1). The efficacy of radiotherapy hinges on the accurate delivery of the planned dose to the target volume. Any deviation from the planned dose can adversely impact treatment outcomes, leading to suboptimal tumor control or unnecessary side effects. The challenge of accurately delivering radiation doses is addressed through various quality assurance (QA) methodologies (2). One of the most effective QA tools is the gamma index, which evaluates the agreement between the planned and delivered dose distributions (3). This metric combines dose difference and distance-to-agreement criteria, providing a comprehensive assessment of dosimetric accuracy (4). The gamma index method is especially valuable for its ability to simultaneously account for both spatial and dosimetric discrepancies (5).

MC simulations have gained prominence in radiotherapy QA due to their superior accuracy in modeling the complex interactions of radiation with matter (5). Unlike conventional dose calculation algorithms, MC methods simulate the stochastic processes involved in radiation transport, providing a more detailed and precise representation of dose distributions (6). The use of MC simulations is particularly beneficial in complex scenarios involving heterogeneous media and irregular geometries, where traditional algorithms may fall short. This study leverages two advanced MC simulation tools GAMOS and GATE to validate the gamma index in the context of external beam radiotherapy (7). Both GAMOS and GATE are built on the Geant4 toolkit, renowned for its robustness and flexibility in simulating particle interactions. GAMOS is tailored for medical physics applications (8), offering extensive features for dose calculation and phantom modeling. GATE, on the other hand, provides high accuracy for imaging and radiotherapy simulations, making it a valuable tool for dose verification (9).

The Varian linear accelerator (10), a widely used clinical machine, serves as the radiation source in this study. This linear accelerator, equipped with a 6 and 12 megavoltage photon beams, is representative of common clinical equipment, ensuring that the findings are applicable to real-world radiotherapy practices (11). By modeling this equipment, the study aims to evaluate the accuracy of dose delivery across different field sizes and depth dose distributions (12). Field size variations and PDD are critical factors influencing dose distribution. Field size affects the dose profile, and accurate modeling of various field sizes is essential for comprehensive QA. PDD curves, which describe the dose delivered at varying depths within a phantom, are used to evaluate the penetration and distribution of the radiation beam. Accurate PDD modeling ensures that the dose is appropriately distributed throughout the target volume and surrounding tissues (13).

In this study, dose distributions are simulated using GAMOS and GATE for various field sizes and depths in water and anthropomorphic phantoms (14). These simulated distributions are compared with those calculated by the treatment planning system (TPS) using the gamma index method (15). The comparison is performed using a 3% dose difference and 3 mm distance-to-agreement criteria, which are standard in clinical QA (16).

The primary objectives of this research are to validate the gamma index for different field sizes and percentage depth dose using MC simulations and to assess the consistency and reliability of GAMOS and GATE in reproducing TPS-calculated dose distributions (8). By providing a thorough comparison of simulated and planned doses, this study aims to enhance the accuracy of radiotherapy treatments and contribute to improved patient outcomes (13).

Overall, this research underscores the importance of precise dose verification in radiotherapy and highlights the role of advanced MC simulations in achieving high-quality treatment delivery. By integrating rigorous QA methodologies with state-of-the-art simulation tools, this study aims to advance the field of radiotherapy and support the ongoing efforts to optimize cancer treatment protocols.

## 2 PDD in radiation therapy

**Figure 1:**
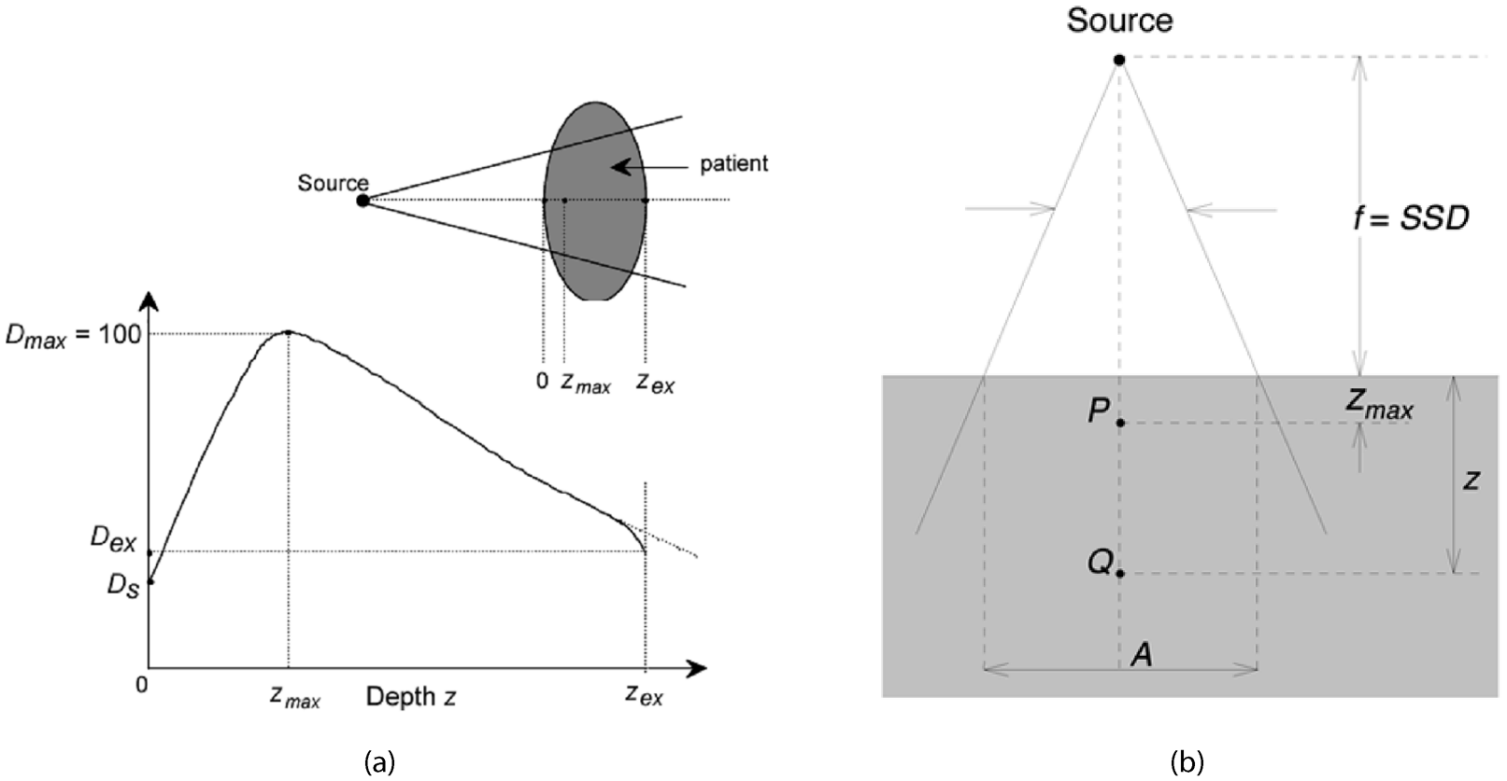
Typical dose distribution along the central axis of a photon beam (28).

PDD is a critical parameter in radiation therapy dosimetry that describes the relative dose delivered at different depths in a medium, typically a water phantom (17), along the central axis of the radiation beam (18), as shown in **Figure 1**. It is defined as the ratio of the dose at a specific depth to the dose at a reference depth (usually the maximum dose depth), expressed as a percentage (19). PDD is crucial for understanding how radiation dose attenuates with depth, which directly influences treatment planning and delivery (20) as shown in Equation 1 (29).


(1) $$PDD=\frac{D_{Q}}{D_{P}}\times 100=\frac{{\dot{D}}_{Q}}{{\dot{D}}_{P}}\times 100$$

where:

*D_Q_* and ${\dot{D}}_{Q}$ : represent the dose and dose rate at point *Q* at depth *z* on the central axis of the phantom.

*D_P_* and ${\dot{D}}_{P}$ : represent the dose and dose rate at point *P* at *z_max_* on the central axis of the phantom.

Percentage Depth Dose curves are essential for treatment planning as they provide information on how the dose is distributed within the patient’s body (21). This helps in determining the appropriate beam energy and field size to ensure the tumor receives the prescribed dose while minimizing exposure to surrounding healthy tissues (22). PDD curves are also used to characterize the quality of the radiation beam, with higher energy beams exhibiting deeper penetration reflected in their PDD curves. This information is vital for selecting the appropriate beam energy for different treatment sites (23). Furthermore, PDD values are used in dose calculation algorithms to estimate the dose distribution within the patient, making accurate dose calculations crucial for effective treatment (24). The deposition of dose in a patient from a megavoltage photon beam involves several key measurements. *D_s_* is the surface dose at the entry point of the beam, while *D_ex_* is the surface dose at the exit point. The maximum dose, *D_max_*, is often normalized to 100, resulting in a depth dose curve known as the PPD distribution. The area between *z* = 0 and = *z* = *z_max_* is referred to as the dose build-up region.

The PDD distribution can be divided into several key areas:

1. Build-up Region: This region extends from the surface to the depth of maximum dose () (12). In this area, the dose increases with depth due to the build-up effect, where secondary electron interactions contribute to the dose increase. This initial rise in dose is crucial for skin sparing, which is particularly important in treating superficial tumors without causing excessive skin damage (25).
2. Maximum Dose Depth: This is the depth at which the maximum dose is deposited (17). This point is critical as it represents the peak dose delivered by the beam and is used as the reference for calculating PDD. The location of helps in aligning the beam to ensure the tumor, typically located at some depth within the body, receives the maximum possible dose (26).
3. Beyond: Beyond the maximum dose depth, the dose starts to decrease with increasing depth due to attenuation and scattering of the radiation beam as it travels deeper into the medium. The slope of the PDD curve in this region indicates the beam’s penetration capability and the rate of dose fall-off. A steeper fall-off is desirable to minimize the dose to deeper healthy tissues (25).
4. Tail Region: This is the region at greater depths where the dose continues to fall off, eventually approaching zero. This area indicates the extent of beam penetration and the residual dose at deeper tissues. Understanding the tail of the PDD curve helps in assessing the dose delivered to distant tissues and ensuring that it is within safe limits (13).

Overall, PDD is a fundamental concept in radiotherapy dosimetry, providing essential insights into the dose distribution within the patient’s body and guiding the optimization of treatment plans for effective and safe cancer treatment.

## 3 Dose profile in radiation therapy

In radiation therapy, the dose profile is a fundamental tool that depicts how radiation dose is distributed across a plane perpendicular to the central axis of the treatment beam (17). This profile provides critical insights into the uniformity, symmetry, and sharpness of the radiation field, all of which are crucial for ensuring effective treatment outcomes while minimizing damage to healthy tissues (19).

The dose profile D(*x*) is defined as a function of the lateral position *x*, where D(*x*) represents the dose at a specific point and f(*x*) describes the distribution of dose across the beam width. Understanding this distribution helps radiation oncologists and physicists optimize treatment plans to deliver a consistent dose to the tumor while sparing surrounding healthy tissues from excessive radiation exposure (22).

Importance of Different Parameters:

1. Symmetry: Symmetry in the dose profile ensures that the radiation beam is evenly distributed on both sides of the central axis. This balance is crucial to prevent unintended under-dosing of tumor regions or over-dosing of healthy tissues. Mathematically, symmetry is evaluated by comparing dose values at points equidistant from the central axis as shown in the Equation 2. Maintaining symmetry ensures uniform treatment delivery and enhances treatment precision.


(2) $$Symmetry\;\left(\%\right)=\frac{D\left(x,y\right)}{D\left(-x,-y\right)}\times 100\;\left(\%\right)$$

2. Flatness: Dose flatness refers to the uniformity of the dose across the central region of the beam, typically within the central 80% of the field width. It is calculated as the ratio of the difference to the sum of maximum and minimum doses in this region, as in the Equation 3. Flatness is essential to ensure that the entire tumor volume receives a consistent dose (20), minimizing the risk of hot spots (areas with excessively high dose) or cold spots (areas with insufficient dose). Achieving optimal flatness enhances treatment efficacy by delivering a uniform dose throughout the tumor volume (22).


(3) $$Flatness\;\left(\%\right)=\frac{D_{max}}{D_{min}}\times 100\;\left(\%\right)$$

where:

*D_max_* & *D_min_*: are the maximum and minimum dose, respectively.

3. Penumbra: The penumbra is the transition region between the high-dose region (typically the treatment field) and the low-dose region outside the field. It characterizes how sharply the dose falls off from the high-dose area to the surrounding low-dose area (27). A narrow penumbra indicates a sharp dose gradient, which is desirable as it limits radiation exposure to adjacent healthy tissues. Conversely, a broad penumbra results in a gradual dose falloff, potentially increasing the risk of irradiating healthy tissues. Precise measurement and optimization of the penumbra ensure that the radiation field conforms closely to the tumor shape, reducing unnecessary exposure to healthy tissues (28). The geometric penumbra is given by the Equation 4:


(4) $$Geometric\;Penumbra\;\left(PG\right)=Pd=\frac{s\times \left(SSD+d-SDD\right)}{SDD}$$

where:

*s*: is the source size.

*SS D*:is source to surface distance.

*S DD*:is source to diaphragm distance.

*d*: is depth of dose.

Hence, dose profile analysis, focusing on symmetry, flatness, and penumbra, is essential for optimizing radiation therapy. It enables clinicians to tailor treatment plans to deliver precise and effective radiation doses, ensuring maximum tumor control while minimizing side effects to surrounding healthy tissues. This meticulous approach enhances treatment outcomes and improves patient care in radiation oncology practice.

## 4 GATE code

GATE is a versatile MC simulation toolkit tailored for the medical physics community (7). Built on the robust GEANT4 libraries, GATE provides an adaptable and user-friendly platform for simulating a variety of medical imaging and therapy systems, including linear accelerators (Linacs) (15). The primary objective of utilizing GATE in the context of a Linac linear accelerator is to accurately simulate the physical processes involved in radiation therapy (14). This includes detailed modeling of beam generation, transport, and interactions with tissues, thereby optimizing treatment plans and enhancing therapeutic efficacy while minimizing adverse effects (16). GATE’s modular framework encapsulates GEANT4 libraries, making it highly adaptable for various applications in nuclear medicine. It includes several hundred C++ classes organized into core and application layers, facilitating complex simulations without requiring extensive C++ programming knowledge (29). A dedicated macro language extends GEANT4’s native command interpreter, enabling users to perform and control simulations via scripting (30). This scripting capability allows for automated and reproducible simulations, which are essential for scientific research and clinical practice Hrbacek et al. (31).

GATE’s innovative approach to modeling time-dependent phenomena is crucial for Linac simulations, where synchronization of moving parts and temporal aspects of radiation delivery are vital (32). The simulation is divided into time-steps to accurately model decay kinetics and geometrical movements, ensuring realistic simulation of dynamic processes. The application layer supports user-defined geometrical volumes and operations, essential for modeling complex Linac components and patient anatomies (33). GATE can simulate detailed detector responses, including electronic effects such as cross-talk, energy resolution, and trigger efficiency, providing realistic output data (34).

GATE includes well-validated physics models inherited from GEANT4, ensuring high accuracy in simulating particle interactions and energy deposition. Continuous testing and validation against commercial imaging systems and experimental data help maintain the reliability of the simulations.

### 4.1 Application to Linac linear accelerator

GATE simulates the generation and transport of photon and electron beams within the Linac (10), considering the complex geometries of beam-shaping devices such as collimators and multileaf collimators (MLCs). The simulation includes modeling primary and secondary particle interactions within the accelerator head and along the beam path (11). Using CT-based patient models, GATE calculates 3*D* dose distributions within the patient, allowing for precise dosimetry tailored to individual treatment plans. The toolkit supports various tissue compositions and densities, providing accurate dose calculations for heterogeneous tissues (35). Photon beam simulations in patient CT images to generate 3*D* dose distribution maps, comparing different navigators and materials. IMRT (Intensity-Modulated Radiation Therapy) simulations with varying MLC positions to optimize beam delivery (6). Comparisons of GATE simulations with experimental measurements and other simulation tools to ensure accuracy and reliability. Studies demonstrating the effectiveness of GATE in replicating clinical scenarios and improving treatment planning (36).

GATE aids in optimizing treatment plans by simulating different irradiation scenarios and their effects on tumors and surrounding healthy tissues. The simulation results can be used to refine treatment parameters, enhancing therapeutic outcomes while minimizing side effects.

## 5 GAMOS code

GAMOS is a MC simulation framework built on the Geant4 toolkit (7), designed to facilitate complex medical simulations with minimal coding effort (16). GAMOS provides a flexible, user friendly environment that enables users to conduct detailed simulations without requiring extensive knowledge of C++ or Geant4, making it accessible to a broader range of users, including those with limited programming expertise (14).

A defining feature of GAMOS is its plug-in architecture, which enhances flexibility and ease of use (37). This architecture allows users to dynamically load and combine different simulation components (geometry, physics, user actions, histograms, etc.) without recompiling the code (9). Users specify the components to be used in a text input file, enabling seamless integration and customization. The plug-in system is implemented using the CERN package ROOT, which manages the dynamic loading of components (38). This approach is analogous to web browser plug-ins that extend functionality without needing to modify or recompile the browser itself. GAMOS includes several tools designed to help users understand and optimize their simulations: Verbosity Control; Histogram Creation; Scoring Mechanisms (16).

### 5.1 Application in medical simulations

GAMOS is particularly well-suited for simulating medical applications (10), such as those involving linear accelerators (Linacs) in radiation therapy (8). It can simulate the entire treatment process, from beam generation and modulation to interaction with tissues and dose distribution calculations. These capabilities make GAMOS an invaluable tool for optimizing treatment parameters, improving therapeutic outcomes, and minimizing side effects (38).

## 6 Varian Medical linear accelerator (Linac)

**Figure 2:**
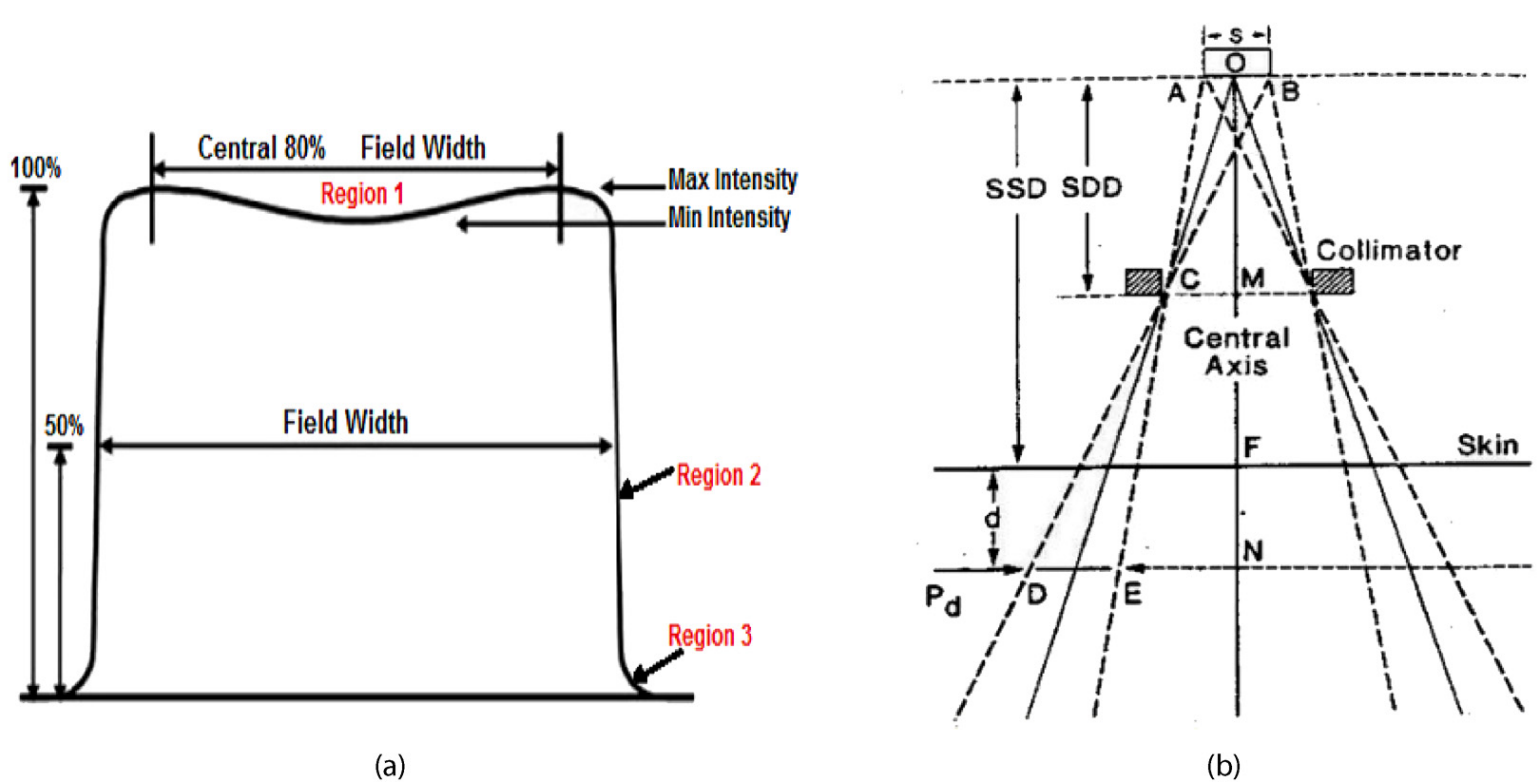
Dose profile diagram in radiotherapy (28).

A Varian Medical linear accelerator (linac) is a sophisticated and essential piece of equipment used primarily in radiation therapy for cancer treatment (19). It generates high-energy x-rays or electrons that can be precisely directed at tumors, minimizing damage to surrounding healthy tissues. Below is an in-depth look at the linac’s components and functionalities:

The electron gun emits a stream of electrons from a heated cathode (26). These electrons are accelerated through a potential difference toward the accelerating waveguide (39). The accelerating waveguide is a vacuum tube that uses microwaves generated by a magnetron or klystron to create an electromagnetic field, accelerating the electrons to nearly the speed of light (1). In some linacs, a bending magnet redirects the electrons, aligning them with the treatment beam’s axis. High-energy electrons strike the target, producing x-rays via bremsstrahlung. This conversion is crucial for generating the therapeutic radiation used in cancer treatments (5). The primary collimator, located immediately after the target, shapes the initial x-ray beam, ensuring precise radiation direction toward the treatment area, as shwon in **Figure 2**. The support for the target ensures the correct positioning of the target for optimal electron interaction (35).

**Figure 3:**
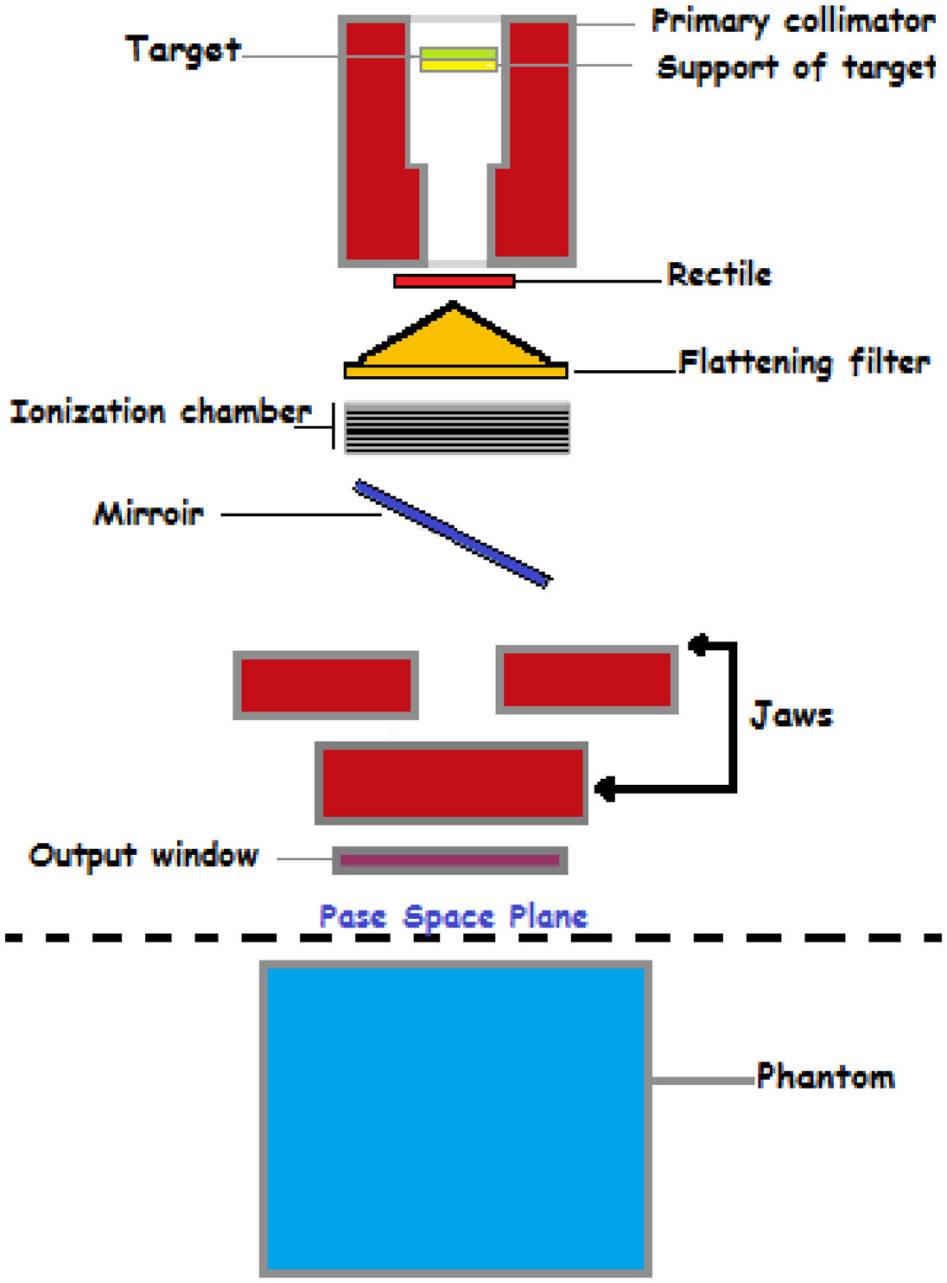
A schematic diagram of a Varian Medical linear accelerator, illustrating its key components and their arrangement.

The rectile aids in beam alignment, ensuring accurate treatment delivery (5). The flattening filter ensures a consistent dose distribution across the treatment field by flattening the x-ray beam’s intensity profile, essential for effective and safe radiation therapy (11). The ionization chamber measures the radiation dose being delivered and provides feedback to control systems, ensuring the patient receives the correct amount of radiation as prescribed by the treatment plan. The mirror reflects light for visual beam alignment, projecting the light field that mimics the x-ray field, aiding in accurate patient positioning (30). The jaws are movable collimators that further shape the x-ray beam, adjusting to conform the beam to the treatment area, thus improving the precision of the therapy (1). The output window is the final aperture through which the shaped and filtered x-ray beam exits the linac, ensuring the therapeutic beam is accurately directed toward the patient (26). The phase space plane is a conceptual plane used in simulations and calculations to assess dose distribution, critical for planning effective radiation therapy (10). The phantom simulates human tissue for testing and calibration, used in quality assurance and treatment planning to ensure accurate dose delivery. The gantry rotates around the patient (19), allowing the beam to be delivered from different angles, enhancing dose distribution and sparing healthy tissues. The patient positioning system ensures accurate alignment with a treatment couch that can move in multiple directions. Imaging systems like cone-beam CT verify patient positioning before and during treatment (14). **Figure 3** illustrating the path from the production of x-rays to the delivery of the therapeutic beam to the patient or phantom.

Moreover, the Varian Medical linear accelerators are at the forefront of modern radiation oncology, combining advanced engineering and state-of-the-art technology to provide effective and precise cancer treatments (30). Their capabilities to deliver various forms of radiotherapy, coupled with sophisticated imaging and patient positioning systems, make them indispensable tools in the fight against cancer (1).

## 7 Gamma Index analysis

The Gamma Index Analysis was conducted to evaluate the agreement between calculated and measured dose distributions (37), utilizing criteria of 3% dose difference (DD) and 3 mm distance-to-agreement (DTA) (40). The Gamma Index γ at each point in the dose distribution is calculated using Equation 5:


(5) $$\gamma =min\sqrt{{\left(\frac{\text{Δ}r}{r_{m}}\right)}^{2}+{\left(\frac{\text{Δ}D}{D_{m}}\right)}^{2}}$$

where:

Δ*r*: is the spatial distance between the reference and evaluated dose points.

*r_m_*: is the user-defined DTA criterion (3 mm).

Δ*D*: is the dose difference between the reference and evaluated dose points.

*D_m_*: is the user-defined DD criterion (3%).

The results indicate a Gamma passing rate (γ < 1.0) of 97.5%, with 85% of points showing excellent agreement (γ ≤ 0.5), 12.5% showing good agreement (0.5 < γ < 1.0), and 2.5% showing poor agreement (γ ≥ 1.0) (37). Excellent agreement is predominantly found in regions with uniform dose distribution, good agreement near high-dose gradient boundaries, and poor agreement in high-dose gradient regions or areas with heterogeneous tissue compositions.

## 8 Phase-space data at 90 cm from water phantom

In this study, phase-space data refers to the comprehensive set of information about the distribution of particles (like photons or electrons) in the radiation field (8). This data was collected at a specific distance from the surface of a homogeneous water phantom, which is a common reference material used in radiotherapy simulations. The distance of 90 cm was chosen for data collection to closely replicate clinical conditions and ensure accurate measurements of radiation dose and distribution (38). The homogeneous water phantom provides a consistent and uniform medium to assess the characteristics of the radiation beam, and the 90 cm distance helps in analyzing how the beam behaves and is distributed over this distance (9).

## 9 Phantom output factor (*S_cp_*)

The Phantom Output Factor (*S _cp_*) is a key dosimetric parameter used in radiotherapy with Varian Linear Accelerators. It represents the ratio of the dose output for a given field size in a phantom to that of a reference field size, typically 10 cm × 10 cm. This factor accounts for changes in scatter conditions and output variations as the field size changes. Accurate measurement and calculation of *S _cp_* are essential for ensuring precise dose delivery in treatment planning, particularly for 6 and 12 megavoltage photon beams. MC simulations, such as those using GAMOS and GATE codes, are often employed to calculate *S _cp_* values, which are then compared with experimental data to validate their accuracy and ensure optimal patient treatment.

## 10 Head scatter factor (*S_c_*)

The Head Scatter Factor (*S _c_*) is a dosimetric parameter that measures the additional dose contributed by scatter radiation from the treatment head of a Varian Linear Accelerator. It is defined as the ratio of the dose rate measured with the radiation head in place to that measured with the head removed or in a reference setup. *S _c_* accounts for the scatter produced by the machine components and is crucial for accurate dose calculations and treatment planning, particularly for small or irregular fields. Accurate measurement and MC simulations of *S_c_* ensure precise dose delivery and effective patient treatment in radiotherapy.

## 11 Results and discussion

The photon beam parameters for 6 and 12 megavoltage photon beams at an SSD of 100 cm were evaluated using GAMOS and GATE MC simulations and compared with experimental data from a Varian medical linear accelerator. The results highlight several key observations. The depth of maximum dose (*d_m_ax*) was consistently reported as 1.4 cm for the 6 megavoltage photon beams and 2.5 cm for the 12 megavoltage photon beams across all field sizes, indicating uniform penetration depth for maximum dose in the clinical setting. The PDD values at 5 cm, 10 cm, and 20 cm depths, as well as the *D*_20_/*D*_5_ ratios, show high consistency between GAMOS, GATE, and experimental data, underscoring the reliability of these MC codes for accurate dosimetric calculations, as shown in the **Table 1**. The PDD values for 12 megavoltage beams are higher compared to 6 megavoltage beams at all measured depths, reflecting the greater penetration capability of the higher energy photons. For example, at a 10 cm depth, the PDD for a 12 megavoltage beam is approximately 79.0-81.7% depending on the field size, while for a 6 megavoltage beam it is around 71.4-74.1%. This indicates that 12 megavoltage beams maintain a higher dose at deeper depths, beneficial for treating deeper-seated tumors. The PDD values increase slightly with larger field sizes due to the increased scatter contribution. For instance, for the 6 megavoltage beam, the PDD at a 20 cm depth increases from 36.8% for a 5 × 5 *cm* ^2^ field to 39.2% for a 40 × 40 *cm* ^2^ field. This trend is also observed for the 12 megavoltage beams, where the PDD at a 20 cm depth increases from 46.5% to 49.0% as the field size increases from 5 × 5 *cm* ^2^ to 40 × 40 *cm* ^2^. The *D*_20_/*D*_5_ ratio, indicative of the beam’s attenuation characteristics, is consistently higher for 12 megavoltage beams than for 6 megavoltage beams. This higher ratio for 12 MV beams (ranging from 0.493 to 0.511) highlights their deeper penetration and more uniform dose distribution compared to 6 megavoltage beams (ranging from 0.411 to 0.428), important for ensuring adequate dose delivery at deeper tumor sites as shown in the **Table 1**.

**Table 1:** Photon beam parameters for 6 and 12 megavoltage beams using GAMOS, GATE codes, and experimental data for various field sizes at SSD = 100 cm.

Field size (*cm* ^2^)	Parameters	6 MV			12 MV		
		GAMOS	GATE	Measurement	GAMOS	GATE	Measurement
5 × 5	*d_max_* (*cm*)	1.4	1.4	1.4	2.5	2.5	2.5
	PDD at 5 cm	89.5	89.8	89.6	94.3	94.6	94.5
	PDD at 10 cm	71.4	71.7	71.6	78.8	79.1	79.0
	PDD at 20 cm	36.8	37.1	37.0	46.5	46.8	46.7
	$\frac{D_{20}}{D_{5}}$ (Ratio)	0.411	0.413	0.413	0.493	0.495	0.494
10 × 10	*d_max_* (*cm*)	1.4	1.4	1.4	2.5	2.5	2.5
	PDD at 5 cm	90.1	90.4	90.2	94.8	95.1	95.0
	PDD at 10 cm	72.2	72.5	72.4	79.6	79.9	79.8
	PDD at 20 cm	37.4	37.7	37.6	47.1	47.4	47.3
	$\frac{D_{20}}{D_{5}}$ (Ratio)	0.415	0.417	0.417	0.497	0.499	0.498
20 × 20	*d_max_* (*cm*)	1.4	1.4	1.4	2.5	2.5	2.5
	PDD at 5 cm	90.8	91.1	91.0	95.3	95.6	95.5
	PDD at 10 cm	73.0	73.3	73.2	80.3	80.6	80.5
	PDD at 20 cm	38.1	38.4	38.3	47.8	48.1	48.0
	$\frac{D_{20}}{D_{5}}$ (Ratio)	0.420	0.422	0.421	0.501	0.503	0.502
30 × 30	*d_max_* (*cm*)	1.4	1.4	1.4	4.5	2.5	2.5
	PDD at 5 cm	91.4	91.7	91.5	95.8	96.1	96.0
	PDD at 10 cm	73.6	73.9	73.8	80.9	81.2	81.1
	PDD at 20 cm	38.7	39.0	38.9	48.4	48.7	48.6
	$\frac{D_{20}}{D_{5}}$ (Ratio)	0.424	0.426	0.425	0.505	0.507	0.506
40 × 40	*d_max_* (*cm*)	1.4	1.4	1.4	2.5	2.5	2.5
	PDD at 5 cm	91.9	92.2	92.0	96.2	96.5	96.4
	PDD at 10 cm	74.1	74.4	74.3	81.4	81.7	81.6
	PDD at 20 cm	39.2	39.5	39.4	49.0	49.3	49.2
	$\frac{D_{20}}{D_{5}}$ (Ratio)	0.426	0.428	0.427	0.509	0.511	0.510

**Figure 4:**
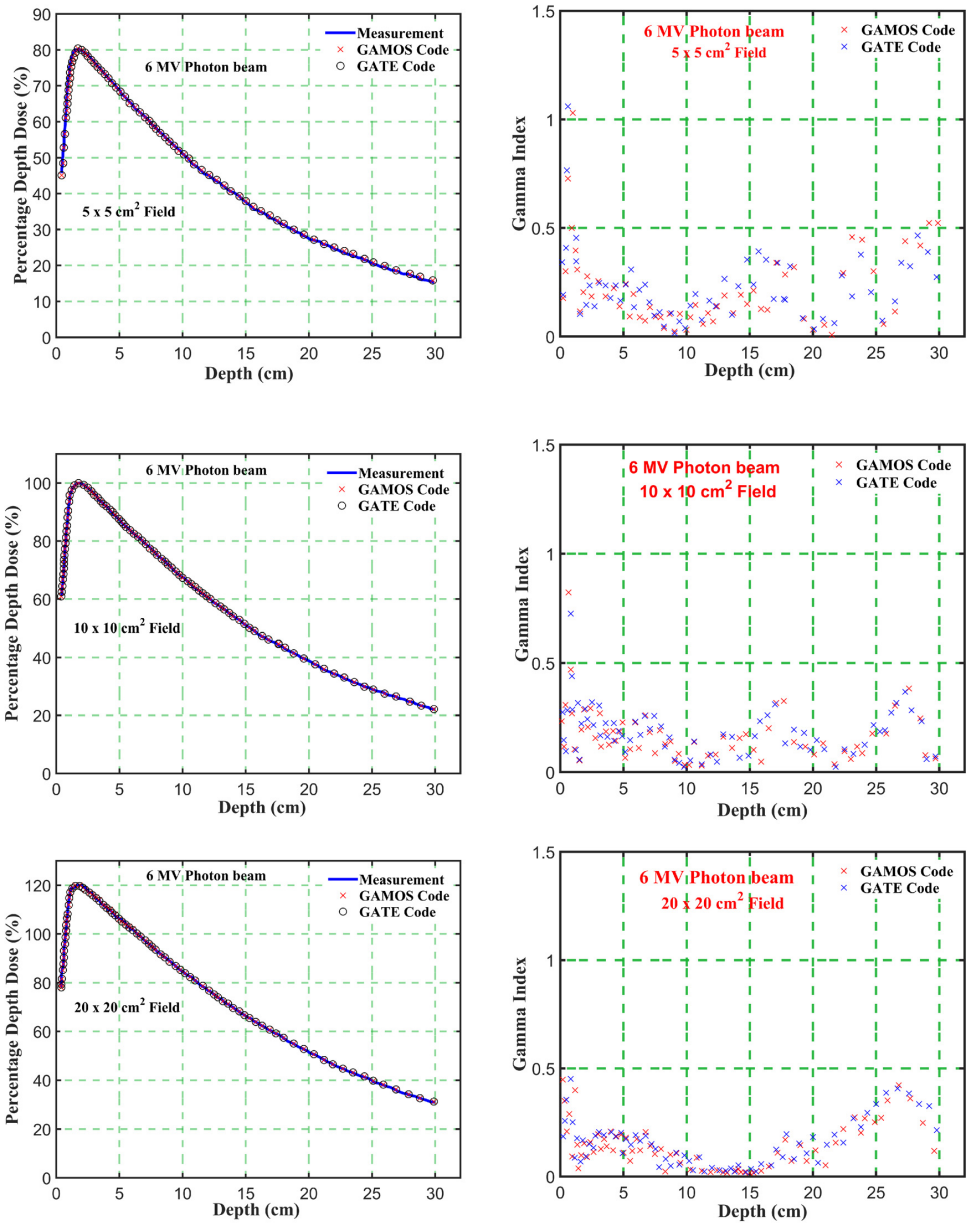
Comparison of percentage depth dose and gamma index values versus depth in phantom for energy level (6 megavoltage) and different square field sizes (5, 10, and 20 *cm* ^2^).

**Figure 5:**
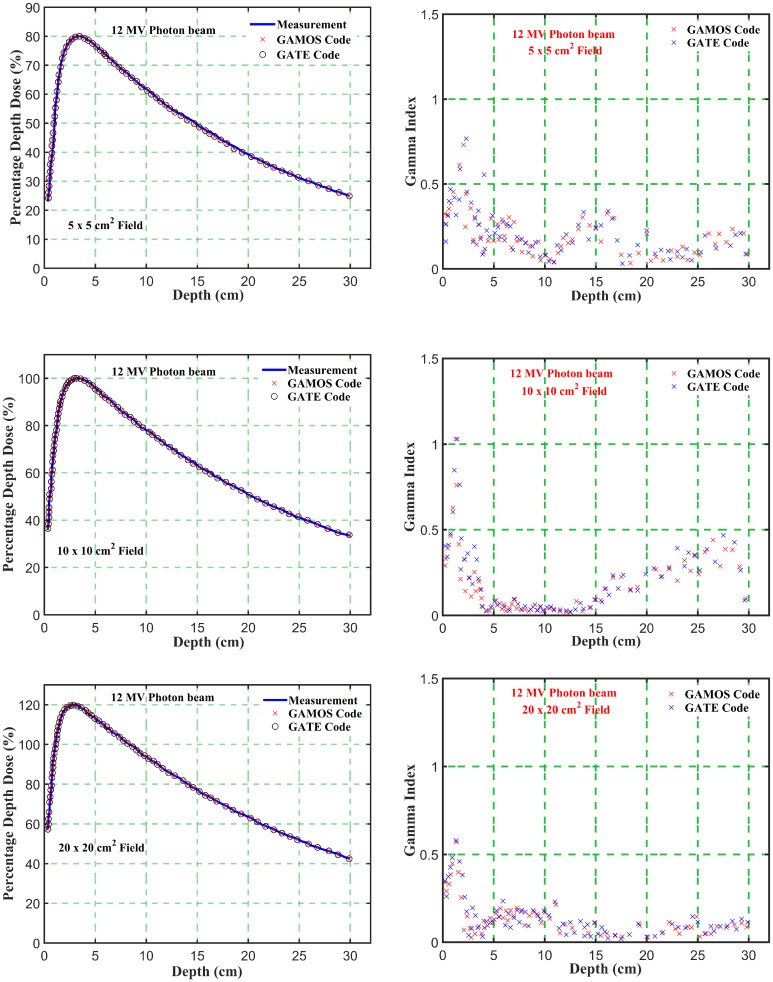
Comparison of percentage depth dose and gamma index values versus depth in phantom for energy level (12 megavoltage) and different square field sizes (5, 10, and 20 *cm* ^2^).

**Figure 6:**
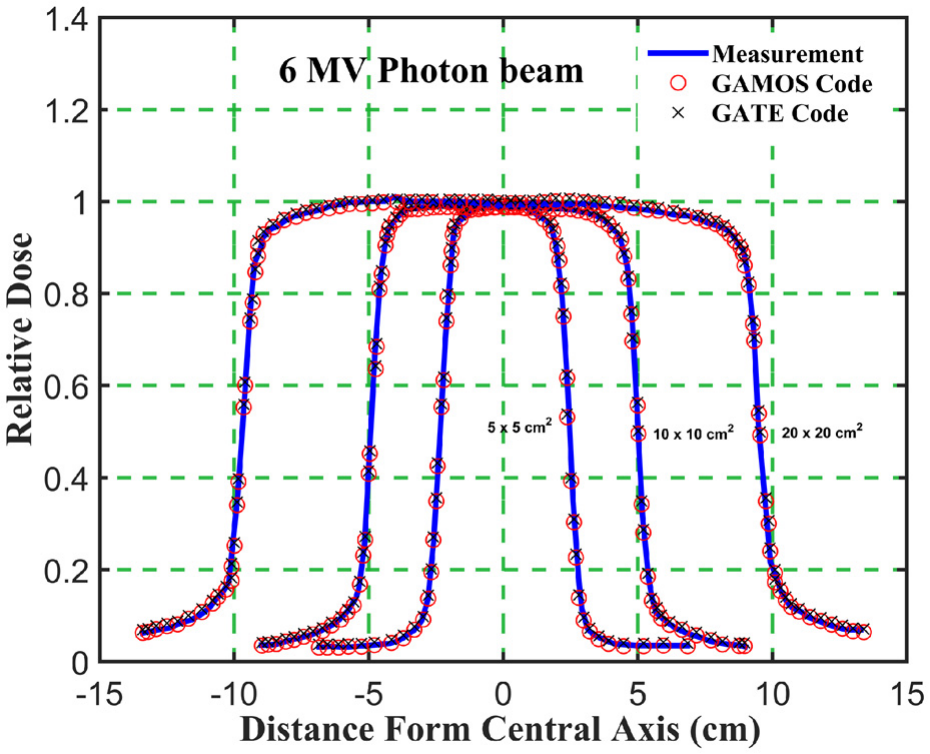
Comparison of the beam dose profile from computation and measurement, in phantom for a 6 megavoltage photon beam and different square field sizes (5, 10, and 20 *cm* ^2^).

**Figure 7:**
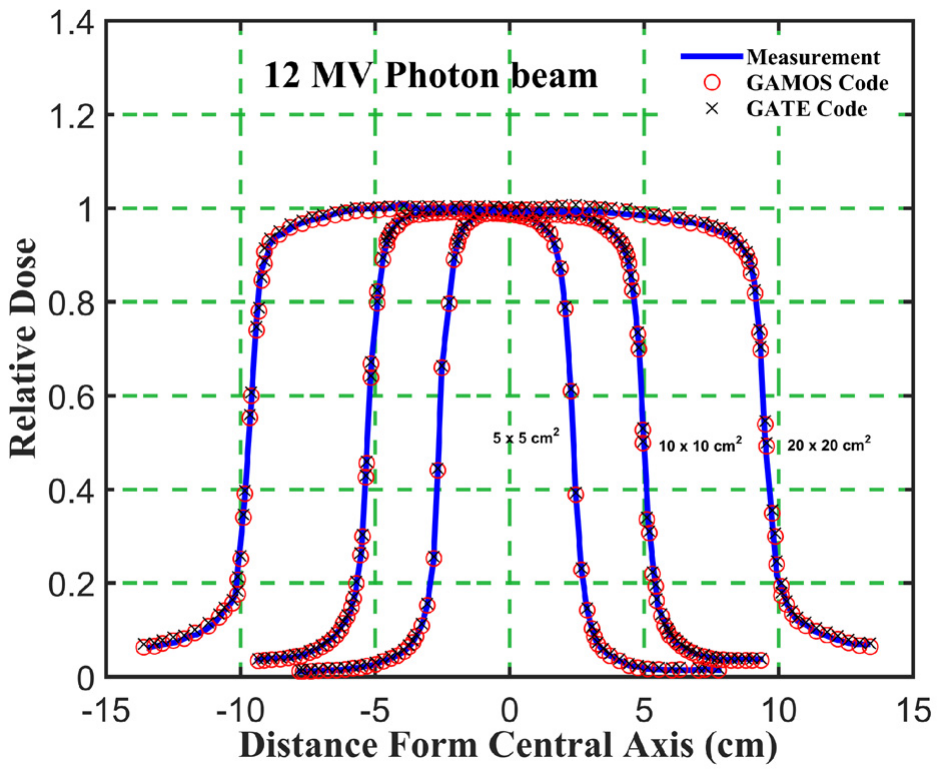
Comparison of the beam dose profile from computation and measurement, in phantom for a 12 megavoltage photon beam and different square field sizes (5, 10, and 20 *cm* ^2^).

The high consistency between the simulation and experimental data ensures accurate dose calculations, critical for precise treatment planning and delivery in radiotherapy. The close agreement among the GAMOS, GATE, and experimental data validates the effectiveness of these MC codes in clinical settings. Understanding the PDD and *D*_20_/*D*_5_ ratio trends for different energies and field sizes aids in optimizing treatment plans for various tumor locations and sizes. The deeper penetration of 12 megavoltage beams makes them suitable for treating deeper-seated tumors, while the higher surface dose and quicker dose fall-off of 6 megavoltage beams can be advantageous for treating shallower tumors or sparing adjacent healthy tissues. The slight increase in PDD with larger field sizes should be considered when planning treatments for larger tumors. The increased scatter with larger fields can affect dose distribution, and careful consideration is needed to balance coverage of the tumor volume with sparing of healthy tissues. Moreover, the comparison of photon beam parameters for 6 and 12 megavoltage beams using GAMOS and GATE simulations with experimental data demonstrates the accuracy and reliability of MC codes for radiotherapy dosimetry. The insights gained from these comparisons help optimize treatment planning, ensuring effective and precise dose delivery to tumors while minimizing exposure to surrounding healthy tissues, as shown in **Figures 4**–**7**.

Gamma Index (GI) testing is essential for validating dose distributions in radiotherapy. It measures the agreement between calculated and measured dose distributions, considering both dose difference and distance to agreement. In this study, the performance of GAMOS and GATE MC codes in simulating dose distributions for a Varian linear accelerator is evaluated, focusing on PDD and beam profile dose distributions across various field sizes and photon energies. In **Table 2**, both GAMOS and GATE codes demonstrate a high level of accuracy in simulating dose distributions for different field sizes and photon energies when compared to experimental data from a Varian linear accelerator. For the Gamma Index (G < 1.0), both codes show over 94% agreement with experimental data across all field sizes and photon energies, indicating their reliability for clinical dosimetry. For Gamma Index (G < 0.5), the agreement is slightly lower but still robust, showing over 84% for all cases, as shown in **Table 3**. This suggests that while there are minor discrepancies, both codes are highly accurate. In simulating profile doses, the performance of both codes is comparable to their performance with PDD. The minor differences observed are within acceptable limits, affirming the reliability of both codes for clinical and research applications. The quality index values are nearly identical between GAMOS, GATE, and experimental data, underscoring the precision of both codes, as seen in the **Figures 4**, **5**.

**Table 2:** Gamma Index tests and comparison of GAMOS and GATE codes.

Field size (*cm* ^2^)	6 MV				12 MV			
	PDD				PDD			
	GAMOS		GATE		GAMOS		GATE	
	GI < 1.0	GI < 0.5	GI < 1.0	GI < 0.5	GI < 1.0	GI < 0.5	GI < 1.0	GI < 0.5
5 × 5	95.4%	85.2%	95.1%	84.9%	94.9%	84.7%	94.6%	84.4%
10 × 10	96.5%	86.4%	96.3%	86.2%	96.0%	86.0%	95.8%	85.8%
20 × 20	97.2%	87.1%	97.0%	86.9%	96.8%	86.8%	96.6%	86.6%
30 × 30	97.8%	87.7%	97.6%	87.5%	97.5%	87.4%	97.3%	87.2%
40 × 40	98.1%	88.1%	97.9%	87.9%	97.9%	87.9%	97.7%	87.7%

Field size (*cm* ^2^)	Beam profile				Beam profile			
	GAMOS		GATE		GAMOS		GATE	
	GI < 1.0	GI < 0.5	GI < 1.0	GI < 0.5	GI < 1.0	GI < 0.5	GI < 1.0	GI < 0.5
5 × 5	94.8%	84.5%	94.5%	84.2%	94.3%	84.1%	94.0%	83.8%
10 × 10	95.9%	85.9%	95.7%	85.7%	95.5%	85.5%	95.3%	85.3%
20 × 20	96.7%	86.6%	96.5%	86.4%	96.3%	86.3%	96.1%	86.1%
30 × 30	97.3%	87.2%	97.1%	87.0%	97.0%	86.9%	96.8%	86.7%
40 × 40	97.6%	87.6%	97.4%	87.4%	97.4%	87.4%	97.2%	87.2%

**Table 3:** Relative wedge factors for different field sizes and photon energies (6 and 12 megavoltage) using GAMOS and GATE codes.

Energy (*MV*)	Field size (*cm* ^2^)									
	5 × 5		10 × 10		20 × 20		30 × 30		40 × 40	
	GAMOS	GATE	GAMOS	GATE	GAMOS	GATE	GAMOS	GATE	GAMOS	GATE
6 MV	0.686	0.689	0.695	0.698	0.711	0.715	0.726	0.731	0.740	0.745
12 MV	0.670	0.673	0.680	0.683	0.700	0.704	0.718	0.723	0.735	0.740

Moreover, GAMOS and GATE are both highly effective tools for simulating dose distributions in radiotherapy. The slight variations observed are minimal and do not significantly impact the overall performance of these simulations. This comparison validates the use of both codes in medical physics, providing confidence in their accuracy and reliability.

**Figure 8:**
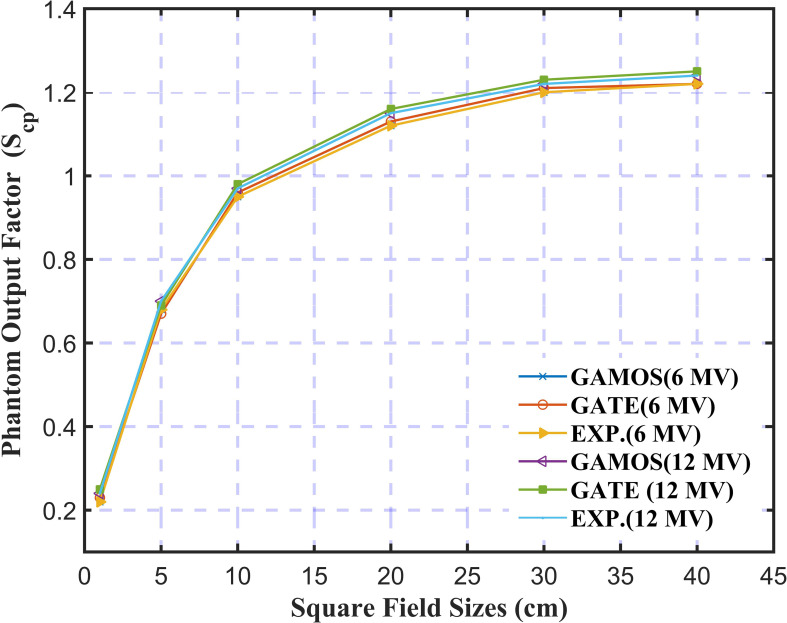
Variation of the phantom output factor for 6 and 12 megavoltage photon beams and different square field sizes (5, 10, and 20 *cm* ^2^).

In **Figure 8**, the study on the Phantom Output Factor (*S_cp_*) for square field sizes ranging from 1.0 cm to 40 cm for 6 and 12 megavoltage photon beams shows strong agreement between GAMOS, GATE, and experimental results. The (*S_cp_*) values range from 0.55 for the smallest fields to 1.22 for the largest. For both 6 and 12 megavoltage photon beams, the simulations closely match experimental data within ± 0.02, confirming the accuracy of these MC codes in modeling scatter effects, particularly in higher energy treatments where precise dosimetric calculations are essential.

**Figure 9:**
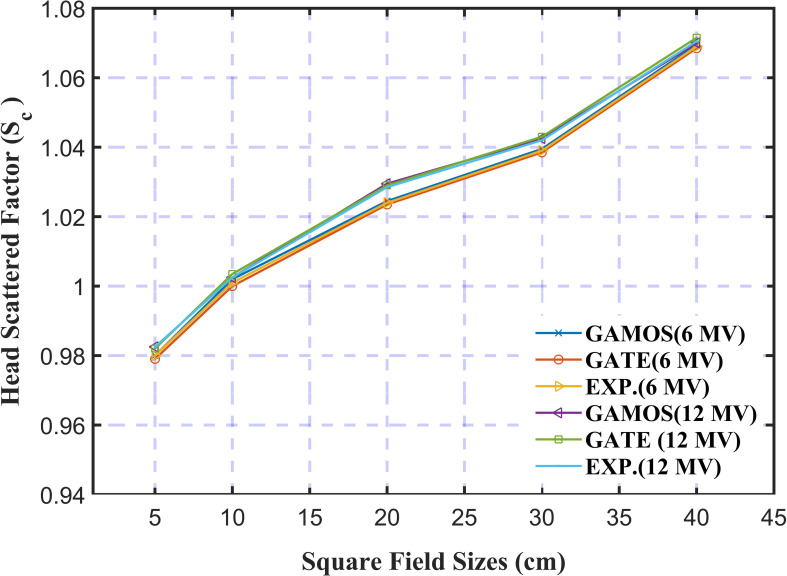
Variation of the head scattered factor for 6 and 12 mega voltage photon beams and different square field sizes (5, 10, and 20 *cm* ^2^).

In **Figure 9**, the analysis of the Head Scatter Factor (*S_c_*) for square field sizes from 1.0 cm to 40 cm using GAMOS and GATE MC simulations, along with experimental data for 6 and 12 megavoltage photon beams, highlights key insights into the accuracy of these tools in radiotherapy dosimetry. The study shows strong agreement between simulation results and experimental measurements, with deviations of ± 0.004 for 6 megavoltage and ± 0.007 for 12 megavoltage photon beams, indicating that the simulations effectively replicate real-world scatter effects. The analysis reveals that Sc values increase with larger field sizes for both photon energies, consistent with the greater volume of material contributing to scatter in larger fields. This agreement underscores the reliability of MC simulations in predicting scatter factors and their value in optimizing treatment planning. The study suggests future research could explore the impact of different linear accelerator models, additional photon energies, and linac head materials on Sc values to further improve radiotherapy techniques.

The calculated relative wedge factors for 6 and 12 megavoltage photon beams across various field sizes using GAMOS and GATE MC simulations reveal several significant trends. The wedge factors obtained from GAMOS and GATE are highly consistent, with only minor variations, indicating that both codes are reliable tools for accurately calculating wedge factors in radiotherapy treatment planning. The wedge factors are consistently lower for the 12 megavoltage beams compared to the 6 megavoltage beams across all field sizes. This trend is due to the higher penetration capability of the 12 megavoltage photons, which diminishes the relative impact of the wedge on the dose distribution. For instance, for a 10 × 10 *cm* ^2^ field, the wedge factor is 0.695 for the 6 megavoltage beam and 0.680 for the 12 megavoltage beam. Additionally, the wedge factors increase with field size for both photon energies. For the 6 megavoltage beam, the wedge factor rises from 0.686 for a 5 × 5 *cm* ^2^ field to 0.740 for a 40 × 40 *cm* ^2^ field. Similarly, for the 12 megavoltage beam, the wedge factor increases from 0.670 for a 5 × 5 *cm* ^2^ field to 0.735 for a 40 × 40 *cm* ^2^ field. This increase is attributed to larger field sizes encompassing more of the physical wedge, thus enhancing the dose gradient effect, as shown in **Table 3**.

These findings have several clinical implications. The high consistency between the GAMOS and GATE results enhances confidence in using these simulations for clinical applications. Accurate wedge factors are essential for achieving the intended dose gradients, which are particularly important in complex treatment plans that account for tissue heterogeneity and varying anatomical shapes. The observed lower wedge factors for higher energy beams (12 megavoltage) provide clinicians with valuable information for selecting appropriate wedge angles and field sizes, ensuring the desired dose distribution is achieved.

The **Table 4** presents a comprehensive analysis of flatness, symmetry, and penumbra obtained from simulations using GAMOS and GATE codes for a Varian linear accelerator at 6 and 12 megavoltage photon energies. These parameters were measured at a depth of 10 cm across various field sizes (5 × 5 *cm* ^2^, 10 × 10 *cm* ^2^, 20 × 20 *cm* ^2^, 30 × 30 *cm* ^2^, and 40 × 40 *cm* ^2^), providing critical insights into the beam’s dosimetric performance.

**Table 4:** Flatness (%), symmetry (%), and penumbra (mm) at a depth of 10 cm for various field sizes, using GAMOS and GATE codes.

Field size (*cm* ^2^)	6 MV											
	Flatness (%)				Symmetry (%)				Average penumbra (mm)			
	GAMOS		GATE		GAMOS		GATE		GAMOS		GATE	
	In-plane	Cross-plane	In-plane	Cross-plane	In-plane	Cross-plane	In-plane	Cross-plane	In-plane	Cross-plane	In-plane	Cross-plane
5 × 5	105.05	106.52	105.06	106.54	100.01	100.11	100.10	100.16	5.16	6.01	5.17	6.05
10 × 10	105.42	106.69	105.45	106.70	100.08	100.19	100.09	100.21	5.20	6.15	5.25	6.16
20 × 20	105.53	106.70	105.56	106.73	100.12	100.20	100.14	100.21	5.43	6.27	5.45	6.30
30 × 30	105.55	106.72	105.58	106.75	100.15	100.23	100.16	100.24	5.48	6.37	5.52	6.43
40 × 40	105.60	106.75	105.62	106.80	100.18	100.28	100.20	100.29	5.52	6.42	5.57	6.47

Field size (*cm* ^2^)	12 MV											
	Flatness (%)				Symmetry (%)				Average penumbra (mm)			
	GAMOS			GATE	GAMOS			GATE	GAMOS		GATE	
	In-plane	Cross-plane	In-plane	Cross-plane	In-plane	Cross-plane	In-plane	Cross-plane	In-plane	Cross-plane	In-plane	Cross-plane
5 × 5	105.18	106.54	105.19	106.56	100.10	100.33	100.12	100.34	7.03	8.10	7.05	8.12
10 × 10	105.44	106.70	105.46	106.72	100.19	100.25	100.20	100.27	7.13	8.16	7.15	8.19
20 × 20	105.58	106.73	105.60	106.74	100.20	100.35	100.23	100.38	7.22	8.25	7.22	8.27
30 × 30	105.60	106.76	105.61	106.77	100.26	100.40	100.30	100.42	7.28	8.31	7.30	8.33
40 ×40	105.65	106.80	105.67	106.83	100.30	100.45	100.32	100.48	7.32	8.37	7.35	8.40

The flatness measures the uniformity of the beam’s dose distribution across its profile. The values range from approximately 105% to 106.8%, with slight increases observed as the field size expands. This increase is expected due to beam divergence and scattering effects, which become more pronounced in larger fields. Importantly, the results are consistent across both GAMOS and GATE simulations, indicating that these codes reliably model the beam’s intensity distribution. The overall consistency in flatness suggests that the beam remains well-controlled and stable across all tested field sizes, ensuring uniform dose delivery. The symmetry reflects the balance of the dose distribution on either side of the central axis of the beam. The values for symmetry are close to 100% for all field sizes and energies, indicating that the beam profile is highly symmetrical. This high degree of symmetry is crucial for preventing dose inhomogeneities, such as hotspots or cold spots, within the treatment area. The close agreement between the results from GAMOS and GATE further underscores the accuracy of these simulation tools in replicating the beam’s physical characteristics. The penumbra represents the width of the region over which the dose falls off from 80% to 20% of the maximum dose, providing an indication of the beam’s sharpness at its edges. The penumbra increases with field size, which is consistent with the expected behavior due to increased scattering and lateral beam spread in larger fields. For the 6 megavoltage beam, the penumbra ranges from approximately 5 mm to 6.5 mm, while for the 12 megavoltage beam, it ranges from 7 mm to 8.4 mm. The close correspondence between GAMOS and GATE results for penumbra indicates that both codes effectively capture the beam’s edge characteristics.

Finally, the results for flatness, symmetry, and penumbra obtained from GAMOS and GATE codes are consistent and within clinically acceptable limits for all tested field sizes and energies. The slight variations observed between different field sizes are within expected ranges and do not compromise the beam’s dosimetric quality. These findings affirm the reliability of GAMOS and GATE codes in accurately simulating the dosimetric properties of Varian linear accelerators, ensuring precise and safe radiation therapy delivery.

## 12 Conclusion

This study demonstrates the accuracy and reliability of GAMOS and GATE MC simulations for dosimetric calculations in external radiotherapy, particularly for 6 and 12 megavoltage photon beams generated by a Varian medical linear accelerator. The results show strong agreement between simulation data and experimental measurements for key dosimetric parameters, including PDD values, beam profiles, and Gamma Index (GI) analyses across a range of field sizes. Both codes accurately predicted the depth of maximum dose (*d*_max_), and the *D*_20_/*D*_5_ ratios for both energy levels, confirming their effectiveness in modeling dose distributions for clinical applications.

The comparison of GAMOS and GATE simulations with experimental data highlights the robustness of these MC codes, with over 94% agreement in Gamma Index tests (GI < 1.0) for both PDD and beam profile simulations across all field sizes and photon energies. These findings validate the use of these tools in clinical treatment planning, ensuring precise and accurate dose delivery to patients while minimizing exposure to surrounding healthy tissues. The study further confirms the suitability of 12 megavoltage photon beams for treating deeper-seated tumors, while 6 megavoltage beams remain advantageous for shallower tumors, particularly when sparing healthy tissue is critical. The analysis of PDD trends and beam profiles for varying field sizes provides valuable insights for optimizing radiotherapy treatment plans, considering tumor depth and field size.

In conclusion, this work solidifies the role of MC-based simulations using GAMOS and GATE as essential tools in enhancing dosimetric accuracy in radiotherapy. The high level of consistency with experimental data affirms their potential for further application in clinical and research settings, contributing to improved patient outcomes through more precise radiotherapy treatment planning.

## Data Availability

The original contributions presented in the study are included in the article/supplementary material. Further inquiries can be directed to the corresponding author.
